# Towards an understanding of physical activity-induced post-exertional malaise: Insights into microvascular alterations and immunometabolic interactions in post-COVID condition and myalgic encephalomyelitis/chronic fatigue syndrome

**DOI:** 10.1007/s15010-024-02386-8

**Published:** 2024-09-06

**Authors:** Simon Haunhorst, Diana Dudziak, Carmen Scheibenbogen, Martina Seifert, Franziska Sotzny, Carsten Finke, Uta Behrends, Konrad Aden, Stefan Schreiber, Dirk Brockmann, Paul Burggraf, Wilhelm Bloch, Claudia Ellert, Anuradha Ramoji, Juergen Popp, Philipp Reuken, Martin Walter, Andreas Stallmach, Christian Puta

**Affiliations:** 1 Department of Sports Medicine and Health Promotion, Friedrich-Schiller-University Jena, Wöllnitzer Straße 42, 07749 Jena, Germany; 2 Center for Interdisciplinary Prevention of Diseases Related to Professional Activities, Jena, Germany; 3 Institute of Immunology, Jena University Hospital/ Friedrich-Schiller-University Jena, Jena, Germany; 4 Institute of Medical Immunology, Charité-Universitätsmedizin Berlin, Corporate Member of Freie Universität Berlin and Humboldt Universität Zu Berlin, and Berlin Institute of Health (BIH), Berlin, Germany; 5 German Center for Cardiovascular Research (DZHK), Partner Site Berlin, Berlin, Germany; 6 Department of Neurology, Charité-Universitätsmedizin Berlin, Corporate Member of Freie Universität Berlin and Humboldt-Universität Zu Berlin, Berlin, Germany; 7 Children’s Hospital, School of Medicine, Technical University of Munich, Munich, Germany; 8 German Center for Infection Research (DZIF), Berlin, Germany; 9 AGV Research Unit Gene Vectors, Helmholtz Munich (HMGU), Munich, Germany; 10 Institute of Clinical Molecular Biology, Kiel University and University Medical Center Schleswig-Holstein, Campus Kiel, Kiel, Germany; 11 Department of Internal Medicine I, Kiel University and University Medical Center Schleswig-Holstein, Campus Kiel, Kiel, Germany; 12 Center Synergy of Systems, TU Dresden University of Technology, Dresden, Germany; 13 mHealth Pioneers GmbH, Körtestraße 10, 10967 Berlin, Germany; 14 Department for Molecular and Cellular Sports Medicine, Institute for Cardiovascular Research and Sports Medicine, German Sport University Cologne, Cologne, Germany; 15 Landarztnetz Lahn-Dill, Wetzlar, Germany; 16 Initiative Long COVID Deutschland, Lemgo, Germany; 17 Institute of Physical Chemistry (IPC) and Abbe Center of Photonics (ACP), Member of the Leibniz Centre for Photonics in Infection Research (LPI), Friedrich-Schiller-University Jena, Jena, Germany; 18 Leibniz Institute of Photonic Technology, Member of Leibniz Health Technologies, Member of the Leibniz Centre for Photonics in Infection Research (LPI), Jena, Germany; 19 Department for Internal Medicine IV (Gastroenterology, Hepatology and Infectious Diseases), Jena University Hospital, Jena, Germany; 20 Department of Psychiatry and Psychotherapy, Jena Center for Mental Health, Jena University Hospital, Jena, Germany; 21 German Center for Mental Health (DZPG), Partner Site Jena, Jena, Germany; 22 Center for Sepsis Control and Care (CSCC), Jena University Hospital/Friedrich-Schiller-University Jena, Jena, Germany

**Keywords:** Post-exertional malaise, Post COVID condition, Physical activity, SARS-CoV-2, ME/CFS

## Abstract

**Background:**

A considerable number of patients who contracted SARS-CoV-2 are affected by persistent multi-systemic symptoms, referred to as Post-COVID Condition (PCC). Post-exertional malaise (PEM) has been recognized as one of the most frequent manifestations of PCC and is a diagnostic criterion of myalgic encephalomyelitis/chronic fatigue syndrome (ME/CFS). Yet, its underlying pathomechanisms remain poorly elucidated.

**Purpose and methods:**

In this review, we describe current evidence indicating that key pathophysiological features of PCC and ME/CFS are involved in physical activity-induced PEM.

**Results:**

Upon physical activity, affected patients exhibit a reduced systemic oxygen extraction and oxidative phosphorylation capacity. Accumulating evidence suggests that these are mediated by dysfunctions in mitochondrial capacities and microcirculation that are maintained by latent immune activation, conjointly impairing peripheral bioenergetics. Aggravating deficits in tissue perfusion and oxygen utilization during activities cause exertional intolerance that are frequently accompanied by tachycardia, dyspnea, early cessation of activity and elicit downstream metabolic effects. The accumulation of molecules such as lactate, reactive oxygen species or prostaglandins might trigger local and systemic immune activation. Subsequent intensification of bioenergetic inflexibilities, muscular ionic disturbances and modulation of central nervous system functions can lead to an exacerbation of existing pathologies and symptoms.

## Introduction

The persistence of long-term symptoms of COVID-19 is a common phenomenon among those who contracted a SARS-CoV-2 infection. A meta-analysis of 31 studies revealed that 43% experience lingering or newly appearing symptoms one month post-infection [1]. Symptoms that persist longer than three months post-infection are subsumed under the term Post-COVID Condition (PCC) [2]. Based on population-based studies from the United States, the number of people suffering from PCC is estimated to be around 6.9% [3]. In this context, it bears noting that acute disease severity has an impact on the risk of developing PCC [4]. Specifically, patients who have been hospitalized are more likely to experience residual symptoms compared to non-hospitalized individuals [5], with asymptomatic cases exhibiting the lowest risk of having PCC [6]. Yet, the mechanisms of PCC may differ following a severe versus mild infection. In addition to that, the risk of persistent symptoms is significantly lower in vaccinated compared to unvaccinated subjects [7, 8]. Whether the risk differs between variants and dependent on medication treatment during acute illness remains controversial [9–13].

The clinical spectrum of PCC comprises a variety of different symptoms affecting multiple organ systems, with fatigue, headache, shortness of breath, cognitive impairment, exercise intolerance and post-exertional malaise (PEM) being among the most commonly reported symptoms [14–18]. The latter refers to an inadequate exacerbation of symptoms and a prolonged recovery phase, most frequently triggered by physical exertion [15]. PEM is a debilitating clinical manifestation and it has been reported that at least 68% of individuals with PCC experience it [15, 19]. Moreover, it is considered a hallmark symptom for the diagnosis of myalgic encephalomyelitis/chronic fatigue syndrome (ME/CFS). Although PEM can occur in other chronic diseases, the severity and length of PEM allows to distinguish ME/CFS from other diseases with similar clinical spectrums such as fibromyalgia or multiple sclerosis [20]. An overlapping clinical picture and the fact that more than one year post-infection 19–58% of PCC patients meet the diagnostic criteria for ME/CFS suggests a common etiology of both syndromes [21–24]. Beyond that, the onset of ME/CFS is most commonly described to be preceded by infection-like episodes [25]. However, the spectrum of PCC is more heterogenous compared to post-acute infection syndromes triggered by other pathogens. Specifically, the severity and duration of PEM in PCC varies, with a subset of PCC patients having episodes that last only for several hours or that are not severe enough to fulfill the Canadian or IOM criteria for ME/CFS [24]. Even though COVID-19 is not more likely to be associated with ME/CFS than other infections [26], this points towards potential virus-specific mechanisms underlying PEM in PCC.

However, there is still no unifying understanding of the pathophysiology of these conditions and the mechanisms that elicit episodes of PEM following acute and regular physical activities. In this review, we aim to conceptualize the evidence that has emerged on PCC and ME/CFS pathophysiology which has improved our understanding of the processes that lead to the development of PEM.

## PEM symptom characterization across the post-acute infection syndrome spectrum

PEM is characterized by a disproportional clinical deterioration of one or multiple symptoms that can occur up to 72 h following exertional activities that were tolerated prior to the illness [27, 28]. This state can last for several days or weeks and is barely alleviated by rest or sleep [15]. The most common trigger of such “crashes” is physical exertion [27]. Other potential triggers include cognitive and emotional exertion, insufficient sleep, temperature extremes or orthostatic stress [29]. Reflecting its proposed pathophysiology and the fact that many patients experience a worsening of immune or nervous system related symptoms [30], PEM is also referred to as post-exertional neuroimmune exhaustion or simply post-exertional symptom exacerbation.

A characteristic decrease in function following physical exertion in patients with ME/CFS has been objectified by studies conducting two-day cardiopulmonary exercise testing (CPET). Patients with ME/CFS declined significantly in measures of aerobic capacity and workload on the second day of testing while controls improved in these performance instances [31]. In line with that, patients with ME/CFS showed greater declines in hand grip strength than healthy controls upon repeated testing, which correlated with higher PEM scores [32].

Importantly, PEM is clinically often accompanied by fatigue and profound exercise intolerance. Yet, while being pathogenically connected, they constitute separate entities of the post-infectious disease manifestations. In fact, exercise intolerance refers to the inability to uphold acute exercise due to lack of energy, or rapid development of palpitations, tachycardia, or breathlessness. On the other hand, PEM describes an inadequate delayed regulatory response that elicits the aggravation of symptoms such as fatigue, pain or cognitive impairment and a decrease in the physical capacity level. Importantly, this can occur after exercise as well as after moderate physical activity and activities of daily living [30]. [25]. In severe ME/CFS, already minor activities such as sitting up or brushing teeth can trigger PEM. For the context of this paper and in accordance with previous definitions, we refer to physical activity as any muscle-induced bodily movement that increases energy expenditure above resting conditions and to exercise as planned and structured forms of physical activity [33].

## Hypoxic metabolic response profile to acute physical activity stimuli

The understanding of physical activity-induced symptom exacerbations requires consideration of the acute metabolic response to exercise stimuli and elicited regulatory processes in affected patients. Among others, findings established by studies conducting CPET using incremental cycle ergometry revealed a response pattern that suggests aberrant cell metabolism under hypoxic environments in PCC. Accordingly, patients with persistent symptoms exhibited a reduced aerobic capacity and attained their anaerobic threshold earlier compared to controls. Specifically, multiple studies reported a lower peak oxygen uptake (VO_2peak_) in PCC patients, regardless of acute disease severity [34–37]. In line with that, a meta-analysis of nine studies with 464 subjects showed that the mean difference in VO_2peak_ of PCC subjects was 4.9 mL/kg/min lower than that of individuals that completely recovered from infection [38]. Correspondingly, an analysis of surrogates of mitochondrial function during exercise testing revealed that in addition to higher blood lactate concentrations, at peak exercise subjects with PCC showed significantly lower levels of β-oxidation of fatty acids compared with control subjects, which might serve as an indication for a premature switch to anaerobic glycolysis [35].

The mechanisms underlying reduced aerobic capacity are still lacking conclusive evidence, with some studies interpreting the observed limitations as muscular deconditioning [39]. Durstenfeld et al. concluded that 80% of studies that attributed deconditioning to diminished exercise capacity included subjects that were previously hospitalized with acute COVID-19 and thus potentially immobilized for extended periods of time [38]. Moreover, there exist studies that included non-hospitalized subjects that exhibited diminished aerobic capacity compared with healthy controls, while physical activity levels before infection and at examination did not differ significantly, which makes the contribution of deconditioning less likely [34]. In line with that, the functional limitations observed in PCC exceed the decline in oxygen uptake that would be expected from bed rest only [40, 41]. Beyond that, although it has to be noted that there is evidence that cardiovascular impairments exist among PCC patients [42, 43], cardiac and ventilatory limitations of exercise performance are rather uncommon [38].

**Fig. 1:**
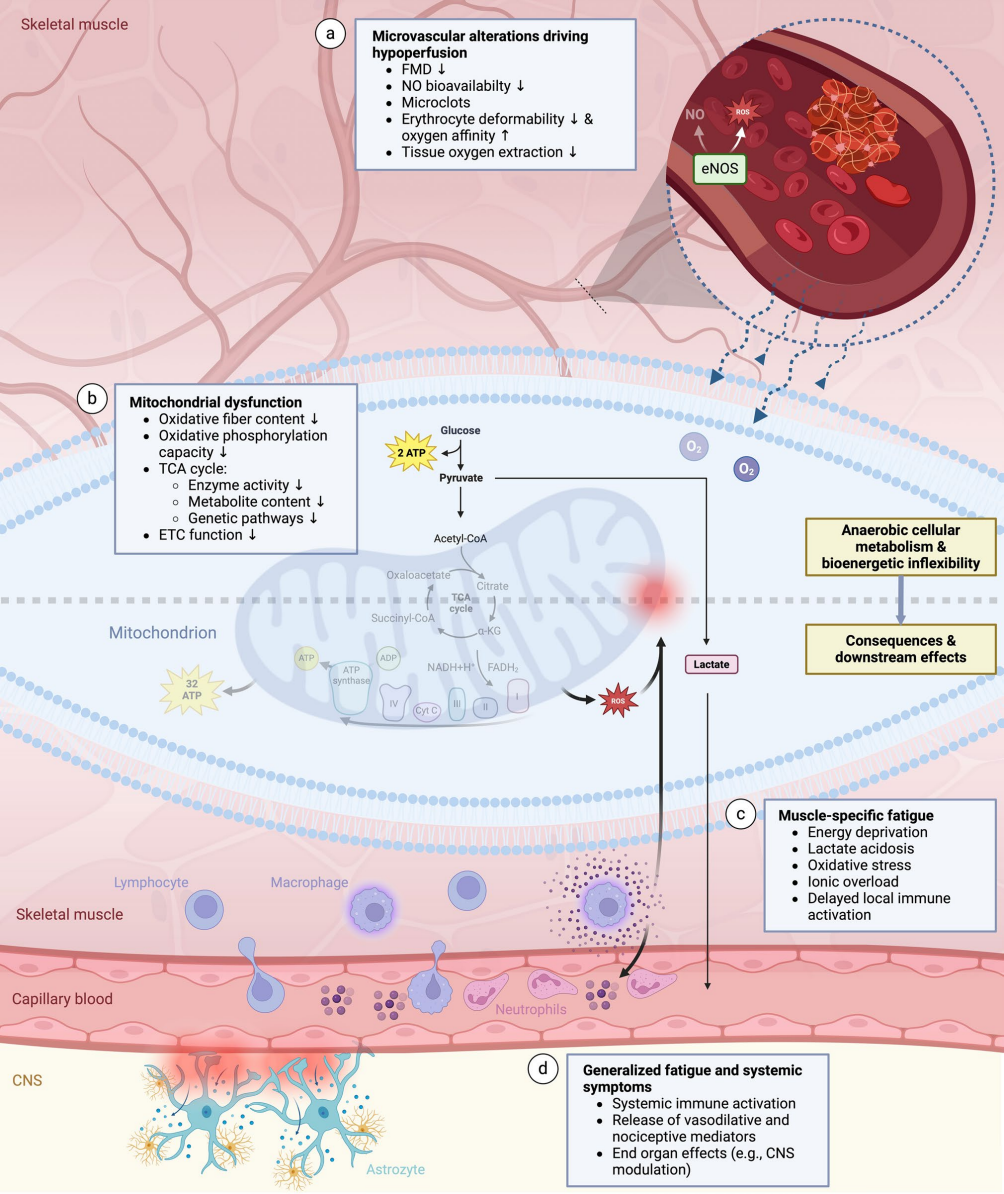
Potential drivers of PEM include microvascular alterations and mitochondriopathy that can functionally culminate in reduced systemic oxygen extraction and oxidative phosphorylation capacity upon physical activities (**a**–**b**). Altered bioenergetics limit the patients’ ability to be physically active and induce the accumulation of lactate, reactive oxygen species and cations (**c**). Overexertion could manifest as delayed symptom exacerbation and systemic fatigue through subsequent immune activation that might aggravate bioenergetic inflexibilities and modulate CNS functions (**d**) (Figure created with biorender.com) *ATP* adenosine triphosphatem, *CNS* central nervous system, *eNOS* endothelial nitric oxide synthase, *ETC* electronic transport chain, *FMD* flow-mediated dilation, *NO* nitric oxide, *ROS* reactive oxygen species, *TCA* tricarboxylic acid

In contrast, more profound analyses gave indications for loss of oxygen transport and metabolism pathway integrity [34, 44] (Fig. 1). Specifically, findings from invasive hemodynamic assessments combined with CPET revealed decreased systemic and peripheral oxygen extraction at peak exercise in PCC subjects which was attributed to reduced oxygen diffusion in the peripheral microcirculation [44–46]. This was particularly supported by findings from a study using near-infrared spectroscopy that revealed a reduced fractional oxygen extraction at the muscular level as well as a lower oxidative capacity [34].

Thus, reduced oxygen extraction is found in both ME/CFS and PCC [47]. Evidence accumulates that mechanisms underlying impairments in peripheral oxygen consumption may include dysregulated microcirculation and mitochondrial dysfunction, as outlined below.

## Muscular mitochondriopathy in PCC and ME/CFS

Evidence that mitochondrial function can be diverted in PCC comes from studies conducting histochemical analyses of muscle biopsy samples. Morphologically, histological staining of vastus lateralis samples revealed a significantly lower percentage of succinate dehydrogenase (SDH) positive and higher percentage of SDH-negative fibers in PCC patients compared to recovered controls [34]. With SDH being an essential enzyme of the tricarboxylic acid (TCA) cycle, these findings point towards decreased content of oxidative and increased content of glycolytic fibers [34], a finding that was also observed in patients specifically experiencing episodes of PEM [48]. Notably, muscle mitochondrial enzyme activity was shown to further decrease one day after the induction of PEM in PCC patients [48]. Further implications for a shift away from oxidative metabolism were provided by the observation that key metabolites of the TCA cycle (i.e. glutamate, α-ketoglutarate, citrate) and the citrate: lactate ratio are lower in skeletal muscle samples of PCC patients as compared to healthy subjects [48] (Fig. 1b).

In line with that, citrate synthase protein and mRNA levels were lower [34] and genetic pathways related to oxidative phosphorylation and cell respiration were downregulated in patients with persistent symptoms [49]. These findings are also reflected by impaired function of mitochondrial complexes. Specifically, high-resolution respirometry revealed a significantly reduced oxygen flux for mitochondrial complex II, and I and II together [34, 50]. Additionally, a loss of cytochrome c oxidase (complex IV) activity has been reported [51] (Fig. 1b). The significant increase of Wiskott-Aldrich Syndrome Protein Family Member 3 (WASF3) protein in muscle cells of ME/CFS patients has been implicated to be a molecular explanation for these functional mitochondrial disruptions [52]. Accordingly, the overexpression of WASF3 induced by stress to the endoplasmic reticulum has been demonstrated to lead to decreases in subunits of complex IV and thus to impair the assembly of supercomplex III_2_ + IV in mouse muscles. This disruption subsequently caused decreased muscular oxidative metabolism, a reduction in maximal running capacity and higher blood lactate levels. Conversely, knocking down WASF3 in myoblasts improved respiration capacity [52].

Beyond that, previously discussed indications for impaired fatty acid oxidation during CPET have been substantiated by a study finding higher levels of plasma carnitine-conjugated and free fatty acids in rest in PCC compared to control subjects [53]. On the other hand, a decrease in plasma acetylcarnitine may be associated with neurocognitive symptoms [54]. A virus-induced shift towards extra-mitochondrial metabolism has been suspected to inhibit antiviral signaling pathways and to promote viral particle formation for the purpose of replication enhancement [53, 55].

In summary, these findings point towards mitochondrial dysregulation with subsequent impairment of function, e.g. oxidative phosphorylation capacity and a switch towards glycolytic pathways (Fig. 1b). Consequently, alterations in mitochondrial structure and function could lead to lower oxygen pressure. This might also explain why PCC patients exhibit slower decline in tissue oxygenation upon occlusion-induced ischemia than subjects without persistent symptoms [56].

## Hemodynamic and microvascular contribution to bioenergetic alterations

Dysfunctional microcirculation and organ perfusion disturbances are proposed to be key features of PCC. Pathogenically, they are supposed to be induced by several interdependent mechanisms that might further compromise a compensation of tissue oxygenation deficits emerging upon exertional activities.

Centrally involved in the development of COVID-19 complications is an endotheliopathy [57] that can outlast the acute illness and is associated with the persistence of symptoms [58]. Correspondingly, PCC patients exhibit elevated levels of circulating endothelial cells, endothelial colony-forming cells, and reduced ADAMTS-13: von Willebrand factor (vWF) ratio, implying ongoing cell damage [59, 60]. Beyond that, a lower flow-mediated dilation (FMD) in patients with PCC suggests a maladaptive capacity of the endothelium to adjust the vascular tone, which has been directly related to symptom persistence [58, 61]. Of note, both a reduced FMD and ADAMTS-13: vWF ratio are additionally associated with a lower exercise capacity, suggesting a potential contribution of endothelial dysfunction to peripheral oxygenation deficits [62, 63]. Of note, a recent MRI study showed that PCC patients exhibit widespread decreased brain oxygen levels in grey and white matter, indicating increased cerebral metabolism [64].

A major cause of compromised endothelial function is proposed to be an inadequate nitric oxide production of the endothelial isoform of nitric oxide synthase (eNOS) that is related to low bioavailability of essential substrates, such as arginine [65, 66]. Consistent with that, it has been shown that endothelial cells cultured in the plasma of ME/CFS patients produce less nitric oxide upon exposure to activating substances than in the presence of healthy control serum [67]. The fact that the supplementation of L-arginine improved FMD, physical performance and perception of effort and fatigue, proves that endothelial cell function might be a central disease pathway [66, 68, 69]. Moreover, potential disturbances in the regulation of molecule release by endothelial cells involved in NO availability and angiogenesis in PCC and ME/CFS compared to healthy donors could also impact the endothelial functionality [70]. Additional structural changes, such as a thickened capillary base membrane might further impair oxygen diffusion into peripheral tissues [51, 71] (Fig. 1a).

Secondly, PCC and ME/CFS are characterized by a thromboinflammatory state [59]. In particular, the discussed endothelial cell dysfunction and immune system activation likely trigger ongoing clotting activity [72]. Specifically, the formation of fibrinolysis-resistant microclots has been documented in PCC patients, which might be related to increased levels of antiplasmin [73] (Fig. 1a). Further analyses revealed that thrombogenicity is also a result of increase in platelet binding capacity that inversely correlated with ADAMTS-13 activity [74]. Of note, research on ME/CFS showed that the increase in clotting proteins (fibrinogen chain proteins FGA and FGB) 15 min after CPET until volitional exhaustion positively correlated with PEM that subjects were experiencing 24 h post-exercise [75]. A dysregulated hemostasis with microthrombi leading to small vessel occlusion could consequently lead to hypoperfusion and ischemia–reperfusion injury, for example to the mitochondrion through sodium and calcium overload [76, 77]. The pathogenic role of ischemia and reperfusion in the development of muscular mitochondriopathy is strengthened by the fact that patients with peripheral arterial occlusive disease present with similar morpho-functional changes like the previously discussed in PCC [78]. Yet, there is no evidence of overt microthrombosis formation from muscle histology studies [48, 50, 51, 71].

Lastly, evidence exists suggesting that perfusion and oxygenation deficits may be linked to altered erythrocyte functional morphology and oxygen affinity. Specifically, reduced MCV and MCH as well as structural membrane damages of erythrocytes that limit cell deformability have been demonstrated in COVID-19 convalescents and ME/CFS patients [79, 80]. Consequences of morpho-functional changes for peripheral oxygen homeostasis remain to be determined but diminished capillary trafficking properties and increased peripheral oxygen affinity are discussed [79, 81].

Further mechanisms underlying impairments in microcirculation may include autonomic dysfunction which may be mediated by sympathetic overactivity, autoantibodies or small fiber neuropathy [82]. Autoantibodies binding to adrenergic and muscarinic acetylcholine receptors were shown to correlate with symptoms of impaired peripheral microcirculation and cognitive impairment [83]. Also, a renin-angiotensin system dysfunction occurs as a consequence of COVID-19 and may result in a functional alteration of ACE2 favoring vasoconstriction [84].

## Dysregulated immune activation imposes allostatic load

There is accumulating evidence suggesting that the etiology of microcirculatory and mitochondrial dysfunctions is, to a significant extent, induced by immunological dysregulation. Certain immune signatures indicating a dysregulated immune response to acute SARS-CoV-2 infection have been associated with the risk of developing PCC [85–89]. In this context, a subsequent persistence of viral antigen might be the pathophysiological link to an ongoing immune activation that distinguishes PCC patients from recovered subjects [86, 90]. Indeed, the persistence of spike protein components and viral RNA has been documented in the circulation and tissue reservoirs in a subset of PCC patients [91, 92] and directly attributed to ongoing antigen-specific cellular immune responses [86]. The latter is for instance indicated by a persistent IFN-γ secretion [87], higher levels of circulating SARS-CoV-2-specific antibodies and exhaustion of antigen-specific T cells [90]. Beyond that, high systemic levels of pro-inflammatory cytokines such as IL-1β, IL-6 and TNF-α relate to activation of coagulation pathways and metabolic disruptions [93]. Equally important, dysfunctional immune activity may also facilitate reactivation of harbored viruses that were previously checked by competent immune surveillance. Correspondingly, PCC patients demonstrate higher antibody responses against viruses such as Epstein-Barr virus [94, 95]. An explanation for a potential reduced capacity to respond to pathogens may be altered immunometabolism. Consistent with the discussed aberrant muscular energy metabolism, blood immune cells exhibit functional aberrations that may similarly alter effector functions. Specifically, compared to healthy controls, immortalized ME/CFS patient-derived lymphoblasts showed mitochondrial deficiency of ATP synthesis due to an isolated complex V inefficiency [96]. Lower ATP-linked respiration rates in PBMCs further underline this energy-generating deficiency [97]. Other studies provide evidence for a metabolic shift in favor of oxidation of fatty acids and protein degradation, as indicated by elevated levels of enzymes and transport proteins involved in these pathways [98, 99], as well as increased utilization of lipids upon activation [99].

In line with these findings, it has been proposed that constant immune activation and the fight against latent viral infections could induce a maladaptive behavioral response to limit energy allocation to processes less important to host survival, leaving no spare resources for e.g. activities of daily living [100].

Beyond that, direct pathogenic effects of the spike protein have been demonstrated. In particular, the induction of vWF, adhesion molecule and pro-inflammatory cytokine production by endothelial cells in an NF-κB and NLRP3 inflammasome-depended fashion promotes endotheliopathy and clotting pathology [101, 102]. Spike-mediated thrombogenicity is also caused by its interaction with fibrin(ogen) and prothrombin [103]. Moreover, a potential pathomechanistic role of spike persistence in a subset has been implied by a report of three PCC cases that experienced a rapid remission of symptoms after treatment with a monoclonal antibody cocktail (casirivimab/imdevimab) directed against the receptor binding domain [104].

## Potential downstream effects of anaerobic metabolism

Under exercise conditions, patients with PCC and ME/CFS exhibit metabolic patterns that imply energy production through anaerobic pathways. The previously described microcirculatory impairments and mitochondrial dysfunction might be a mechanistic explanation for the disturbances in peripheral oxygen delivery and utilization.

Remarkably, the discussed metabolic alterations are characterized by elevated levels of lactate in rest and upon exercise [93, 105]. Additionally, anaerobic metabolism causes a deprivation of cellular energy sources, as pyruvate can no longer be oxidized in the TCA cycle and is thus converted to lactate at the expense of ATP that would otherwise be produced via mitochondrial respiration. As a result, local lactate acidosis and energy deprivation likely cause exercise intolerance and demand early cessation. Equally important, in conjunction with other accumulating products of mitochondrial dysfunction, lactate initiates a cascade of downstream effects. Evidence implying its significance in PEM comes for instance from an investigation that showed that ME/CFS patients with elevated lactate levels (≥ 2 mmol/L at rest) were more likely to experience severe PEM than those with normal levels [106].

Beyond that, cellular oxygen deprivation and mitochondrial dysfunction promotes the formation of reactive oxygen species (ROS) and vasodilatory tissue mediators (e.g., prostaglandins, bradykinin, adenosine) [76]. Mechanistically, this could be linked to PEM development by local and systemic immune activation, as the aforementioned substances possess diverse immunomodulatory effects. Specifically, there exists a vicious cycle between redox imbalance, inflammation, and mitochondrial dysfunction in which higher levels of ROS caused by mitochondria and eNOS induce cell damage and immune activation that in turn impair cell organelle function [107, 108]. Similarly, lactate can induce cytokine secretion, cell migration and nuclear translocation of NF-κB subunits [109]. In this context, it bears noting that physical exercise per se stimulates immunomodulation via metabolic and neuroendocrine pathways [110, 111]. From the described evidence it can be assumed that in PCC and ME/CFS, the threshold of activation is shifted in a way that activities of daily living are sufficient to trigger these pathways in some patients. Meanwhile, latent baseline immune activity and dysfunctional oxygenation reduce the regulatory window of homeostatic adaption.

The induction of PEM may include local and systemic mechanisms, reflecting that PEM has been proposed to be composed of muscle-specific fatigue and generalized systemic fatigue as two different experiences [112]. On the skeletal muscle level, the accumulation of immunoregulatory substances by the intensification of metabolic dyshomeostasis during physical activity may cause immune cell recruitment and local activation of inflammatory pathways that aggravate mitochondrial dysfunction [113]. For instance, NF-κB reduces muscle oxidative capacity [34], which may further decrease activity tolerance in the hours or days following activity. As a further consequence of hypoperfusion and mitochondrial dysfunction, the ionic homeostasis in muscles is severely impaired, leading to sodium and calcium overload and secondary muscle and mitochondrial damage [114, 115] (Fig. 1c). This mechanism can explain the development of delayed and prolonged symptom exacerbation and disease aggravation upon repeated PEM (detailed in [115]).

Neurological symptoms of PCC have been proposed to be associated with a blood–brain barrier (BBB) dysfunction that enables extravasation of blood components into the brain tissue [116]. In line with that, recent evidence demonstrated that brain fog is associated with increased BBB permeability, likely driven by systemic inflammation [117]. The spillover of cytokines and chemokines from tissues into the systemic circulation amid exertional activities may correspondingly contribute to the experience of generalized fatigue and aggravation of neurological symptoms due to a modulation of central nervous system (CNS) functions that resembles sickness behavior or overtraining syndrome [100, 118] (Fig. 1d). Accordingly, it has been reported in ME/CFS that the severity of symptom flare after moderate exercise is linked to cytokine activity [119]. Beyond that, it has been demonstrated that exosome-associated mitochondrial DNA that significantly increases after exercise in ME/CFS patients and potentially reaches the CNS by crossing a disrupted BBB stimulates cultured human microglia to secrete IL-1β [120].

## Potential treatment options targeting PEM pathophysiology

Reflecting the still evolving understanding of their pathophysiology, the treatment of ME/CFS and PCC is currently limited to the management of individual symptoms. There is a paucity of causative treatment options for both conditions in general and for PEM specifically. In this context, pacing has been promoted to prevent the aggravation of bioenergetic capacities. This includes to avoid overexertion, allow adequate recovery periods, divide activities of daily living into smaller tasks that can be spread out over the day and to rest before symptoms arise [93, 121]. Beyond that, the discussed mechanisms may provide new starting points for therapeutic trials (Table 1). In particular, investigating options to improve microcirculation may prove to be pivotal to ensure adequate adaption of blood supply during physical activities. This could include the use of vasodilators and NO substrates to improve FMD as well as immunoadsorption to deplete vasoactive autoantibodies.

**Table 1:** Potential treatment options targeting proposed drivers of PEM

PEM pathophysiology	Treatment targets	Potential treatment options	Reference
Microvascular alterations	Flow-mediated dilation and nitric oxide synthase	Substrate and cofactor supplementation (e.g., L-arginine) and molecular modulation Vasodilators (e.g., nebivolol, sildenafil)	[66, 68, 69, 122], NCT00598585
	Fibrin amyloid microclots and platelet pathology	Anticoagulation	[123]
	Vasoactive autoantibodies	Immunoadsorption Apheresis	[124, 125]
	Sympathetic overactivity	Parasympathetic activation (e.g., meditation, breathing techniques)	[126]
Dysregulated immune activation	Viral antigen component persistence Latent viral infections Pro-inflammatory pathways T cell exhaustion	Antiviral therapies (e.g., Paxlovid, Ritonavir, Temelimab) Anti-inflammatory drugs (e.g., corticosteroids, antihistamines) Immunomodulators (e.g., kinase inhibitors, rintatolimod)	[104, 127–130], NCT05576662, NCT05497089
Mitochondrial dysfunction	Respiratory complex function Oxidative and inflammatory stress	Redox balancer (e.g., vitamin E, glutathione, ubiquinol, NADH, selenium) Supplementation of magnesium	[131–133]

Just as important is the consideration of the bidirectional relationship between mitochondrial and immunometabolic alterations in pursuit of new therapeutic targets. Wang et al. demonstrated that alleviating stress to the endoplasmic reticulum decreased WASF3 and thus improved mitochondrial respiration [52]. Yet, they did not investigate how these molecular improvements related to clinical symptoms. It can be suspected that long-term improvements in oxidative capacity can only be achieved when immune activation and oxidative stress is concomitantly reduced. Hence, it may additionally be effective to look further into treatment options targeting viral reservoirs and inflammatory pathways. On the whole, several treatment candidates already proved to be effective in some regards (Table 1). However, more randomized controlled studies with clinically relevant endpoints are necessary, as case studies and case series have yielded contradictory results and do not provide sufficient evidence for individual use outside of studies.

## Conclusions

In this literature review we discuss evidence that several homeostatic functions and regulatory mechanisms that are involved in physiological adaption to exercise are dysfunctional in patients experiencing PEM in PCC and ME/CFS. The accumulation of lactate, ROS, and the deprivation of cellular energy sources upon increased metabolic demand contributes significantly to lower exercise capacity. The complex dynamics of immunometabolic downstream effects may also lead to delayed and prolonged symptom exacerbations and dysregulated recovery. In particular, the disturbed metabolic homeostasis and consecutive ionic imbalance can lead to secondary muscle and mitochondrial damage and immune activation. Hence, exceeding their already reduced activity capacities enters affected patients into a recurrent and self-propagating loop. Considering the results of this review, it bears noting that we narratively synthesized and contextualized the results of multiple separate studies with different research focuses. There has not been a study that provided conclusive evidence for one disease etiology, which means that the described pathophysiological observations do not necessarily coexist. For that reason, future studies should look deeper into pathophysiological connections between herein highlighted systems, such as immunometabolic signatures that are associated with the development of PEM. Beyond that, activity prescriptions should take the pathophysiological mechanisms of PCC and ME/CFS into account to attenuate the risk of provoking PEM.

## Data Availability

No datasets were generated or analysed during the current study.
